# Guidelines on the use of disinfectants: comparison between Malaysia and other countries

**DOI:** 10.3205/dgkh000420

**Published:** 2022-10-12

**Authors:** Su Peng Chua, Mohd Hasni Ja’afar, Kon Ken Wong, Roszita Ibrahim, Wan Nur Nafisah Wan Yahya

**Affiliations:** 1 Department of Community Health, Faculty of Medicine, National University of Malaysia, Kuala Lumpur, Malaysia; 2 Department of Medical Microbiology & Immunology, Faculty of Medicine, National University of Malaysia, Kuala Lumpur, Malaysia; 3 Department of Internal Medicine, Faculty of Medicine, National University of Malaysia, Kuala Lumpur, Malaysia

**Keywords:** policies, guidelines, disinfectant use, comparison, Malaysia

## Abstract

**Aim:**

Sanitation and cleanliness are essential factors in reducing the spread of pathogens and preventing healthcare-associated infections. Disinfectants are associated with better hygiene outcomes to reduce pathogen transmission risk and minimize risks to healthcare workers (HCWs) and patients.

**Methods:**

A literature search was undertaken using the electronic databases Scopus, Web of Science, Ovid and Google Scholar. The inclusion criteria for this study are observational and original research studies dating from the five-year period 2017–2021. Other inclusion criteria are full text, English language, qualitative or quantitative studies relevant to the research question. The exclusion criteria are animal studies, systematic reviews, conference proceedings, abstracts, projection modelling studies, in-vivo or in-vitro studies, and books.

**Results:**

Five study nations included the United States of America (USA), the United Kingdom (UK), China, India and South Korea, together with Malaysia. These nations have existing policies, regulations and guidelines regarding the use of disinfectants. HCWs should be aware of the national laws and guidelines that govern the purchase, distribution and use of disinfectants. They should also understand the different roles of the agencies involved, so the context for the guidance provided is clear. Coordination and collaboration across various stakeholders are required for creating solid policies.

**Conclusion:**

Product research and innovation are indispensable, as appropriate personal protective equipment and safety measures for HCWs and patients have top priority in every nation. Hence, clear guidelines for handling disinfectants, in addition to health education about scientific-evidence-based disinfectants, are required.

## Zusammenfassung

**Zielsetzung:**

Hygiene und Sauberkeit sind wesentliche Faktoren, um die Ausbreitung von Krankheitserregern zu unterbinden und Healthcare-assoziierte Infektionen (HAI) zu verhindern. Der Einsatz von Desinfektionsmitteln erfolgt mit dem Ziel, das Risiko der Übertragung von Krankheitserregern zu verringern und dadurch Infektionsrisiken für Beschäftigte im Gesundheitswesen und für Patienten zu minimieren.

**Methode:**

Es wurde eine Literaturrecherche in den elektronischen Datenbanken Scopus, Web of Science, Ovid und Google Scholar durchgeführt. Die Einschlusskriterien für die Studie sind Beobachtungs- und Originalstudien aus den letzten fünf Jahren (2017–2021). Weitere Einschlusskriterien sind englischsprachige, qualitative oder quantitative Studien im Volltext, die für die Forschungsfrage relevant sind. Ausschlusskriterien sind Tierstudien, systematische Übersichten, Konferenzberichte, Zusammenfassungen, Projektionsmodellierungsstudien, In-vivo- oder In-vitro-Studien und Bücher.

**Ergebnisse:**

Zu den fünf Studienländern gehören die Vereinigten Staaten von Amerika (USA), das Vereinigte Königreich (UK), China, Indien, Südkorea und Malaysia. In diesen Ländern gibt es Strategien, Vorschriften und Richtlinien zur Verwendung von Desinfektionsmitteln. Die Mitarbeiter des Gesundheitswesens sollten die nationalen Gesetze und Richtlinien kennen, die den Kauf, die Verteilung und die Verwendung von Desinfektionsmitteln regeln. Sie sollten auch die verschiedenen Rollen der beteiligten Stellen verstehen, damit der Kontext für die bereitgestellten Leitlinien klar ist. Für die Entwicklung solider Strategien sind Koordination und Zusammenarbeit zwischen den verschiedenen Interessengruppen erforderlich.

**Schlussfolgerung:**

Produktforschung und -innovation sind unentbehrlich, weil eine angemessene persönliche Schutzausrüstung und Sicherheitsmaßnahmen für das Gesundheitspersonal und die Patienten für jede Nation höchste Priorität haben. Daher sind klare Leitlinien für den Umgang mit Desinfektionsmitteln sowie eine wissenschaftlich fundierte Gesundheitsaufklärung über Desinfektionsmittel erforderlich.

## Introduction

Sanitation and cleanliness are essential factors in reducing the spread of bacteria and preventing hospital infections [1]. Healthcare-associated infections (HAI) are a major risk within the healthcare environment. The pathogens that cause these infections are often multi-drug resistant organisms (MRO). These MROs survive in biofilms found on environmental surfaces, and can remain viable for transmission for up to several years [2]. Therefore, disinfection and effective cleanliness management in hospitals are crucial in implementing infection control strategies. In addition, disinfection is used to mitigate the risk of pathogen transmission via fomites and other surfaces within the hospital setting [3]. However, not many guidelines and protocols exist for the selection of disinfectants in the healthcare setting. There is limited guidance on effective surface disinfection against these pathogens. On 3^rd^ March 2020, the US Environmental Protection Agency (USEPA) released List N, a comprehensive list of disinfectants that qualify for use against emerging pathogens such as SARS-CoV-2 [4]. List N includes more than 400 unique products with 33 different types of active ingredients. While this list appears extensive, it lacks the guidance and discussion that must be taken into consideration during the pandemic. Key issues such as efficacy, safety profile, availability and practicality must be addressed. Hence, the objective of this study was to assess and determine the guidelines on the use of disinfecting agents in Malaysia as compared to other nations.

## Methods

### Literature search

The formulation of the research question for this study was based on PICO [5], a tool that assists authors in developing a relevant research question for the review. It is based on three main concepts: Population or Problem, Interest, Comparison and Outcome. Based on these concepts, we included the three main aspects in the review, namely Malaysia (Population), use of disinfecting agents (Interest), other nations (Comparison), and guidelines (Outcome), which guided the authors in formulating the main research question “What are the guidelines on the use of disinfecting agents in Malaysia as compared to other nations?”

### Information sources and search strategy

Four online databases (Scopus, Web of Science, OVID and Google Scholar) were searched from 1^st^ to 31^st ^October 2021 using specific keywords and strategies, including Medical Subject Headings (MeSH) (Table 1 (Tab. 1)). As the search terms (keywords) define the limits and the nature of the literature search, these should be established in a comprehensive way to permit selection of all the related articles, and at the same time, eliminate those that are not relevant. Therefore, the key concepts are transformed into keywords, choosing only the most specific terms. Thesaurus systems such as the MeSH terms of the National Library of Medicine were referred to for selecting the appropriate keywords directly related to the topic of interest.

### Selection Criteria

Defining the inclusion and exclusion criteria for literature selection helps focus on the relevance of the studies to the topic. The inclusion criteria for this study are observational and original research studies dating from the five-year period 2017–2021. Other inclusion criteria are full text, English language, qualitative or quantitative studies articles relevant to the research question. The exclusion criteria are animal studies, systematic reviews, conference proceedings, abstracts, projection modelling studies, in-vivo or in-vitro studies and books. 

### Screening and data collection process

The researchers started the review by formulating appropriate research questions. Next, online search results were exported into EndNote 20.1, and duplicates were removed from the initial search results. Articles were then screened by title, abstract, and full text in Microsoft Excel 2016 based on the exclusion criteria described below for the initial screening. In addition, titles that suggested the study would be related to the policies and regulations of disinfectant usage were included for abstract screening. Information was extracted during the abstract screening to refine inclusion criteria for full-text screening. In cases where it was unclear from the abstract whether a study was eligible for full-text screening, it was included for determination during full-text screening. To increase consistency among reviewers, all reviewers performed the screening process, and if there were any disputes, a decision was made after discussions and reaching a consensus. 

## Results

From the final articles included in the study, five other nations were identified: the United States of America (USA), the United Kingdom (UK), China, India, South Korea and Malaysia. The publication year ranges from 2017 to 2021. Two nations were high-income countries (USA, UK), while the remaining nations were low to middle-income countries (Malaysia, China, India and South Korea). The study designs involved were cross-sectional and qualitative studies. The comparison of Malaysia and other countries together with the agencies involved and the existing policies and regulations for disinfecting agents is depicted in Table 2 (Tab. 2).

Malaysia has a guideline, “Policies and Procedures on Infection Prevention and Control (3^rd^ Edition 2019)”, governed by the Ministry of Health (MOH), Department of Environment (DOE) and National Pharmaceutical Regulatory Agency (NPRA). The permitted surface disinfectants and new disinfectant entities are listed by NPRA [6]. Meanwhile, in the USA, the Healthcare Infection Control Practices Advisory Committee (HICPAC), Food & Drug Administration (FDA), and the Environmental Protection Agency (EPA) govern the use of disinfecting agents. In addition, List N of disinfectants is guided by the Food, Drug and Cosmetic Act and the Guideline for Disinfection and Sterilization in Healthcare Facilities (2008) [7], [8]. 

In the UK, the agency involved is the Health Security Agency guided by the Health and Safety at Work Act (1974) and the Food Standard Agency’s (FSA) guidelines. These two policies and regulations oversee the combined detergent disinfectant solution at a dilution of 1,000 parts per million available chlorine (ppm av.cl.) (HSE [9]). The National Health Commission of China established the China Disinfectant Regulations (2014) to regulate the China Hygienic Disinfectants, which are divided into three categories:

1. imported disinfectants,
2. domestic disinfectants, and
3. new disinfection products [10].

In India, the Food & Drugs Administration (FDA) governs the usage of disinfecting agents under Item 12 in Schedule K. They are guided by the Drugs and Cosmetic Act (1940) and the Essential Commodities Act [11]. On the other hand, the agency responsible for the use of disinfecting agents in South Korea is the Korean Drug Administration. They are guided by the Korean Biocidal Products Regulation (K-BPR) and the Pharmaceutical Affairs Law. There are four categories, namely

1. disinfectants,
2. pest control drugs,
3. preservatives, and
4. other biocidal products, which are monitored by these policies and regulations [12].

## Discussion

During the early stages of the COVID-19 pandemic crisis, there were no approved antiviral agents or drugs available for protection against this deadly disease. However, the remaining effective mitigating strategy was to reduce the transmission of the virus through droplets or close contact [13]. Several guidelines and strategies have been suggested to prevent and control the disease at these levels: the case-related population and the general population. These include the maintenance of hand hygiene, disinfection of surfaces, and adherence to basic cough etiquette [14], [15]. Similarly, the National Health Commission of China (NHCC) issued disinfection protocols for the elderly and rural populations that can be applied worldwide [16], [17]. The World Health Organization (WHO) also suggested guidelines for the reduction of viral load through the cleaning and disinfection of surfaces and wastes with the help of disinfectants, such as 0.1% sodium hypochlorite, 0.5% hydrogen peroxide, or 62%–71% ethanol [18], [19]. However, only a small group of HCWs were aware of the use of 1000 mL chlorine-containing disinfectant for cleaning the walls, floors, and dental premises.

On the contrary, based on the previous studies [20], [21] related to Severe Acute Respiratory Syndrome Corona Virus (SARS-CoV) and Middle East Respiratory Syndrome Corona Virus (MERS-COV), the WHO recommended handwashing with soap and water for 20 seconds followed by alcohol-based hand rub (ABHR) for visibly soiled hands [22]. We also found that the participants lacked knowledge related to the stability of COVID-19 on different surfaces. Researchers reported that COVID-19 remained stable on inanimate surfaces for up to nine days; on tissue or printing papers for up to three hours; on wood and clothes for up to two days; on smooth surfaces, e.g., glass and banknotes, for four days; on stainless steel, the inner surface of surgical masks, and plastic for seven days; and on the outer layer of surgical masks for more than seven days [23], [24].

Meanwhile in Europe, consideration of biocidal products is regulated by Regulation (EU) 528/2012 Biocidal Products Regulation (BPR). They are then divided into four main categories, and disinfectants fall into the first main group “Disinfectants and General Biocidal Products”. The EU Regulation employs a two-step registration procedure for biocidal products which consists of the approval of the substance and biocidal product. The European Chemicals Agency (ECHA) is the main European regulatory authority for implementing the enforcement and legislation of biocidal products [25]. Each of the 28 EU member nations has its own “Evaluation Competent Authority” that is responsible for product authorisation. This review program will be completed by the end of 2024 and approval is based on European efficacy test standards (CEN) for disinfectants and antiseptics.

Before utilizing the guidelines provided, healthcare workers (HCWs) should be aware of the federal laws and regulations that govern the sale, distribution and use of disinfectants. HCWs need to know the requirements pertaining to them when they purchase and use these products. They should also understand the different roles of the agencies, so the context for the guidance provided is clear. For instance, the EPA (USA) requires manufacturers of disinfectants to test formulations by using accepted methods for microbiocidal activity, stability, and toxicity to animals and humans. These manufacturers submit all relevant data to the EPA along with proposed labelling. Then, the EPA concludes the product can be used without causing “unreasonable adverse effects” [8]. After the product and its labelling are registered, the manufacturer can sell and distribute it in the USA. Generally, the EPA regulates disinfectants used on environmental surfaces, not those used on critical or semi-critical medical devices; the FDA regulates the latter. In June 1993, the FDA and EPA issued a “Memorandum of Understanding” that divided responsibility for reviewing and surveillance of chemical germicides between the two agencies. Under the agreement, the FDA regulates liquid chemical sterilants used on critical and semi-critical devices, and the EPA regulates disinfectants used on non-critical surfaces and gaseous sterilants. The FDA published its final guidance document on product submissions and labelling. Antiseptics are considered antimicrobial drugs used on living tissue and thus are regulated by the FDA under the Food, Drug and Cosmetic Act. The FDA regulates liquid chemical sterilants and high-level disinfectants intended to process critical and semi-critical devices. At the CDC (USA), the mission of the Coordinating Center for Infectious Diseases is to guide the public on how to prevent and respond to infectious diseases both in healthcare settings and at home. With respect to disinfectants, part of the CDC’s role is to inform the public (in this case, healthcare personnel) of current scientific evidence pertaining to these products, comment on their safety and efficacy, and recommend which chemicals might be most appropriate or effective for specific microorganisms and settings [7].

The management of HAI is exacerbated by rising rates of antimicrobial resistance (AMR). Healthcare workers and a contaminated hospital environment are increasingly implicated in the transmission and persistence of MRO and other pathogens, such as *Clostridium difficile*. This has resulted in a timely focus on a range of HAI preventive measures. Core components include antimicrobial stewardship to reduce overuse and ensure evidence-based antimicrobial use, infection prevention strategies to control MRO – particularly methicillin-resistant *Staphylococcus aureus* (MRSA), vancomycin-resistant *Enterococcus spp*. (VRE) and, more recently, multi-resistant Gram-negative bacteria. In addition, other prevention strategies include enhanced institutional investment in hand hygiene, hospital cleaning and disinfection, and the development of prescription guidelines and standards of care. AMR surveillance and comparisons of prescription practices are beneficial feedback activities once effectively communicated to end-users. Successful implementation of these strategies requires cultural shifts at the local hospital level and, to tackle the serious threat posed by AMR, greater coordination at a national level. HAI prevention needs to be multi-modal, with broader healthcare collaboration, and the strong support and accountability of all medical staff [2].

The use of disinfectants preferred by cleaning staff has been associated with better hygiene outcomes, mainly where strong chlorine-based disinfectants are the alternative. Therefore, it is important to select appropriate cleaning and disinfectant products to reduce the risk of pathogen transmission and minimize any additional risks to staff and patients. The core considerations for this hospital were minimized safety/health risk to staff members, pathogen transmission-risk reduction, cleaning workload reduction, and a decrease in cost. While using a chlorine-based disinfectant, an assessment by an occupational hygienist in a case study indicated that the reported reactions were related to the strong chlorine odor of the disinfectant. Staff were immediately reassigned, while several required worker-compensation leave before re-deployment. Safe work measures were implemented by limiting chlorine disinfectants or using an alternative disinfectant while on duty [3].

Different countries have different disinfection guidelines based on infrastructure and economic factors. The COVID-19 pandemic is an impetus for these countries to develop newer and more concrete disinfection policies. The costs of production, as well as advantages and disadvantages of each disinfectant vary depending on the type of formulation, surface types and areas of use, such as healthcare, institutional and residential. Contact lead time (minutes) is another critical factor for manufacturing these disinfectants. Hence, research and development is required for emerging pathogen viral claim of effectiveness.

## Study strengths and limitations

This study's strength is that it included a comprehensive search of four major databases. It also included grey literature and considered clinical trials, negative results and newspaper articles. There was also a broad search using general MeSH term headings. The limitation of this study is the exclusion of mathematical and projection modelling studies. The articles were also restricted to English language publications only. Many of the included studies were from Asia, which comprises non-English speaking nations. This study could have missed the wealth of related literature published in other native languages. 

## Conclusions

Coordination and collaboration across various stakeholders are required for creating a policy for disinfectants. Product research and innovation are a must, as appropriate personal protective equipment and safety measures for HCWs are a top priority for every country. All stakeholders and policymakers are urged to follow not only the correct procedures for choosing and using disinfectants, but also WHO guidelines to manage HCWs properly with proper communication and accountability. Proper hand hygiene is one of the essential control strategies, as it irrefutably lowers the likelihood of direct or indirect transmissions of microorganisms. It is vital to select alcohol-based hand sanitizers (ABHS) with the appropriate amount of alcohol and practice the correct hand hygiene technique when cleaning hands to ensure all the microorganisms are effectively killed. Hence, clear guidelines for handling disinfectants in addition to health education about scientific-evidence-based disinfectants are required.

## Notes

### Acknowledgement

The authors would like to acknowledge and thank the Department of Community Health, Faculty of Medicine at University Kebangsaan Malaysia (UKM) for supporting this research. 

### Ethical Statement

This research was ethically approved under the UKM MREC code JEP-2021-487. 

### Funding

There were no grants or funding for this research from the public, private, or non-profit sectors.

### Competing interests

The authors declare that they have no competing interests.

## Figures and Tables

**Table 1:**
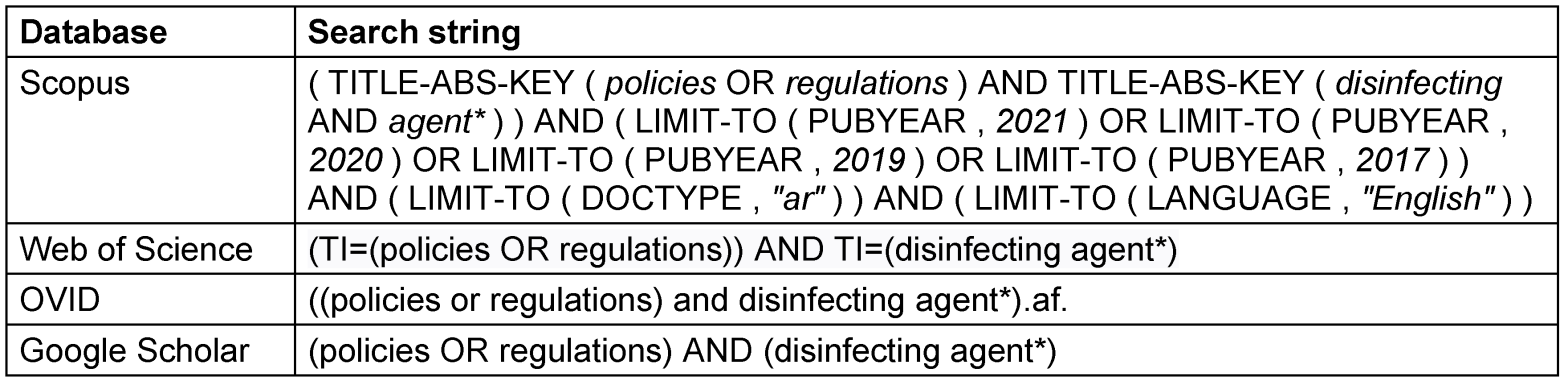
Search string used in the four databases

**Table 2:**
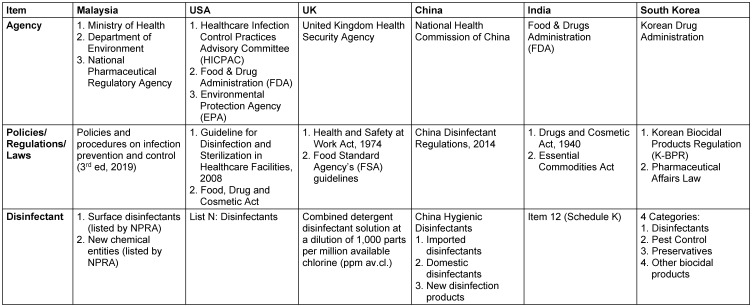
Comparison of agencies according to the articles screened.
